# Hypoxia-Driven Pulmonary Adaptation in the Yak: A Homeostatic Mechanism Mediated by Cell Adhesion Molecules

**DOI:** 10.3390/ijms27031368

**Published:** 2026-01-29

**Authors:** Huizhen Wang, Nating Huang, Xun Zhang, Jingqing Ma, Xiaorong Liu, Jiarui Chen, Qing Wei

**Affiliations:** 1 College of Eco-Environmental Engineering, Qinghai University, Xining 810016, China; ys240713000084@qhu.edu.cn (H.W.); tw202601@outlook.com (N.H.); ys230713000117@qhu.edu.cn (X.Z.); ys230713000158@qhu.edu.cn (J.M.); 230710040119@qhu.edu.cn (X.L.); jrchen@qhu.edu.cn (J.C.); 2 State Key Laboratory of Plateau Ecology and Agriculture, Qinghai University, Xining 810016, China

**Keywords:** cell adhesion molecules, yak lung tissue, adaptation to high-altitude environments

## Abstract

Cell adhesion molecules (CAMs) are key regulators of tissue structural integrity and functional coordination, yet their specific role in the adaptation of yak lung tissue to high-altitude hypoxia remains unelucidated. Thus, we employed transcriptomic sequencing (RNA-seq), molecular biology assays, and single-cell RNA-seq (scRNA-seq) to analyze the expression characteristics of CAMs in yak lung tissues at high and low altitudes. Trypsin or collagenase digestion showed higher cell counts in high-altitude yak lungs (*p* < 0.05). RNA-seq analysis revealed significant enrichment of differentially expressed genes (DEGs) in adhesion-related pathways. Inductively coupled plasma mass spectrometry detected elevated Ca^2+^ levels in high-altitude yak lungs (*p* < 0.05). Quantitative real-time PCR (qRT-PCR) detection of key genes from five major families of CAMs revealed the downregulation of cadherin and integrin family-related genes, and upregulation of immunoglobulin superfamily-related genes, in high-altitude yak lungs (*p* < 0.05), corroborated by immunohistochemical (IHC) staining. A 10× scRNA-seq revealed adhesion changes in 9 of 15 lung cell subpopulations, with differentially expressed CAMs involving integrins. This study demonstrates that yak lung tissue establishes a sophisticated adhesive homeostasis through differential CAMs regulation. This strategy optimizes pulmonary immune responses and energy allocation, ensures structural integrity and functional coordination, and thereby facilitates superior acclimatization to higher-altitude hypoxia.

## 1. Introduction

The Qinghai–Tibet Plateau is known as the ‘roof of the world’. Its unique high-altitude topography has created extreme environmental conditions, including low pressure, hypoxia, cold temperatures, and strong ultraviolet radiation, which pose serious challenges to animal survival [1]. Through long-term natural selection, indigenous animals on the plateau have evolved unique adaptive mechanisms to this extreme environment, with yak (*Bos grunniens*) exhibiting remarkable adaptive capabilities [2,3]. Yaks have a long history of living on the plateau; archeological and genetic evidence indicates that wild yaks (*Bos mutus*) have inhabited the region for at least 2.5 million years, while domestic yaks were domesticated approximately 7300 years ago, with the domestication center possibly located in the eastern Qinghai–Tibet Plateau [4,5]. As a typical plateau-adapted species, the yak exhibits excellent adaptations to the plateau environment across morphological traits, physiological functions, and molecular mechanisms. The unique tongue structure broadens its food sources, the dense fur ensures effective insulation, the strong limbs adapt to complex terrain, and the well-developed cardiopulmonary system significantly improves oxygen uptake, blood circulation, and energy metabolism efficiency, while the upregulated expression of related genes further optimizes these organ functions [6,7,8]. These adaptive traits establish yaks as the ideal model for investigating plateau adaptation in mammals.

Hypoxia is recognized as the most prominent characteristic of plateau environments, while sufficient and stable oxygen supply is essential for maintaining cellular functions and mammalian survival [9]. As a key sensory organ, the yak’s lung tissue responds first to plateau hypoxia stress by initiating multi-level adaptive regulatory mechanisms [7]. Compared with lowland cattle breeds, yaks have larger lungs with greater alveolar surface area, a more developed pulmonary capillary network, a thinner blood–air barrier, and stronger vasodilatory and vasoconstrictive capacity in pulmonary arteries, all of which significantly enhance their oxygen uptake and utilization efficiency [10,11,12]. Research has identified that hypoxia-adaptive related genes (e.g., *HIF-1α*, *HO-1*, *HYOU1*, and *BMPR2*) are significantly enriched in yak lung tissues and maintain pulmonary function by regulating cell survival, angiogenesis, antioxidant defense, and inflammatory response [13,14]. The normal physiological function of lung tissue depends on both cell–cell and cell–extracellular matrix (ECM) interactions, which are mediated by a protein family called CAMs [15]. CAMs are transmembrane glycoproteins located on the cell surface and can be classified into five major families based on their structural characteristics: the cadherin family, the immunoglobulin superfamily, the selectin family, the integrin family, and other adhesion molecules [16,17]. Cadherins ensure vascular permeability and material exchange efficiency in lung tissue through maintaining endothelial cell junctions and modulating ubiquitination [18]. As an immunoglobulin superfamily member, VCAM-1 regulates immune cell trafficking and recruitment by mediating specific adhesion between leukocytes and vascular endothelial cells, thereby facilitating repair at pulmonary injury sites [19]. The constitutive expression of P-selectin in lung tissue promotes the selective adhesion and directed migration of lymphocytes, participating in immune surveillance and host defense processes in the lung [20]. The expression of integrin family member ITGB4 ensures appropriate flexibility and normal ECM composition in lung tissue, supporting healthy lung tissue development [21]. As fundamental regulatory components of multicellular life systems, CAMs precisely coordinate cell–cell interaction networks and play central regulatory roles in both tissue morphogenesis and homeostasis maintenance [15,22,23].

In summary, CAMs are important molecules that respond to chronic hypoxia stress during the plateau adaptation of yak lung tissue, and their dynamic expression is an important factor in maintaining lung tissue homeostasis. However, the expression differences in CAMs in yak lung tissues across different altitudes and their adaptive roles in high-altitude environments remain unclear. Therefore, this study utilized yak lung tissues from high- and low-altitude regions as research objects to comparatively analyze the differential expression of CAMs, to further identify key adhesion-associated cell types and their specifically expressed adhesion molecules, and to elucidate the regulatory role of distinct CAMs expression patterns in hypoxic adaptation of yak lungs, ultimately providing a theoretical basis for understanding high-altitude environmental adaptation in yaks.

## 2. Results

### 2.1. The Result of Cell-Counting

**Figure 1:**
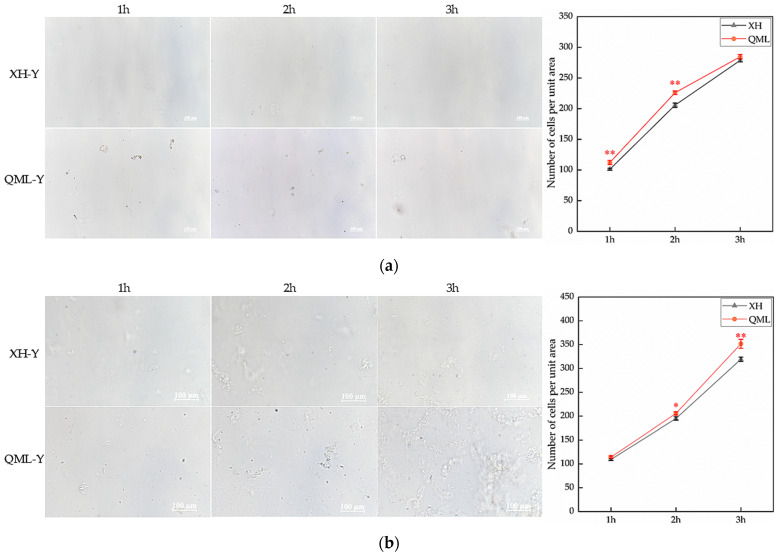
Cell counts from enzymatic digestion of high- and low-altitude yak lung tissues: (**a**) 0.25% trypsin digestion and cell-counting results, Scale bar = 100 μm; (**b**) 2 mg/mL type I collagenase digestion and cell-counting results. Asterisks indicate statistically significant differences between groups (*, *p* < 0.05; **, *p* < 0.01). XH, Xunhua (low-altitude control group); QML, Qumalai (high-altitude experimental group).

High- and low-altitude yak lung tissues were digested with 0.25% trypsin and 2 mg/mL type I collagenase, and cell counts from both enzymatic digestions were performed at 1 h, 2 h, and 3 h post-digestion. The trypsin-digested group exhibited highly significant differences in cell numbers at 1 h and 2 h, with significantly more cells isolated from high-altitude yak lung tissue compared to low-altitude yaks (*p* < 0.01) (Figure 1a). The group digested with type I collagenase exhibited significant differences in cell numbers at 2 h and 3 h, with significantly more cells isolated from high-altitude yak lung tissue than from low-altitude yaks (*p* < 0.05) (Figure 1b).

### 2.2. DEGs and Enrichment Analysis Results

**Figure 2:**
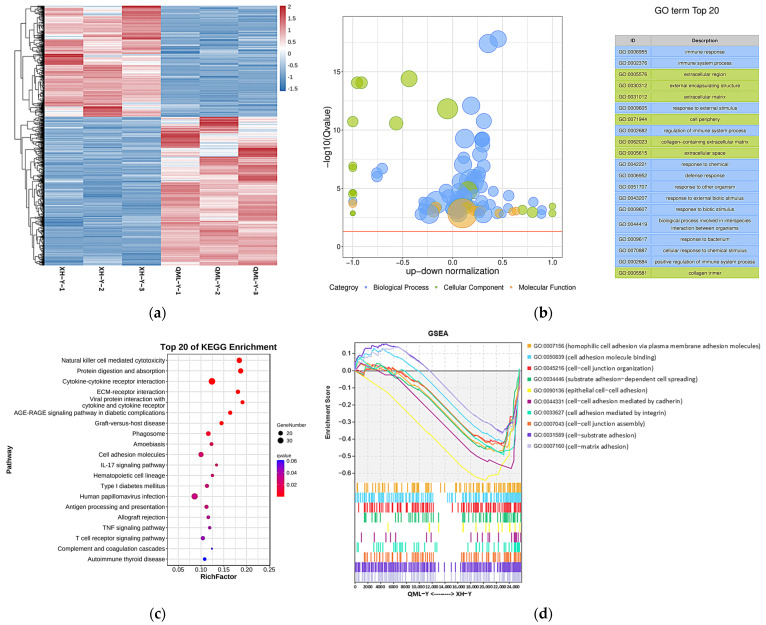
DEGs and their functional analysis results in high- and low-altitude yak lung tissues. (**a**) Clustering heatmap of DEGs, where red indicates upregulated genes and blue indicates downregulated genes; (**b**) GO enrichment results of DEGs. −log_10_(Q-value) on the *y*-axis indicates significance; the *x*-axis shows the proportion of upregulated (positive) and downregulated (negative) genes; GO categories: blue represents biological process, green represents cellular component, and orange represents molecular function; the yellow line marks the Q-value = 0.05 threshold; (**c**) KEGG enrichment bubble chart of DEGs, where dot size represents the number of genes enriched in each pathway, and redder color indicates smaller *p*-value (i.e., higher pathway significance); (**d**) GSEA results of adhesion-related GO terms. The *x*-axis indicates gene rank in the list, while the *y*-axis ‘Enrichment Score’ reflects the enrichment degree of each gene set (higher scores indicate more significant enrichment), with positive values representing activated states and negative values indicating suppressed states.

After analyzing the RNA-seq data, a total of 1139 DEGs were identified between high- and low-altitude yak lung tissues, including 652 upregulated and 487 downregulated genes. Clustering analysis showed that DEGs clustered distinctly between high- and low-altitude yak lung tissues (Figure 2a). GO and KEGG enrichment analysis results showed that these DEGs were mainly enriched in GO terms including immune response (GO: 0006955), immune system process (GO: 0002376), extracellular region (GO: 0005576), external encapsulating structure (GO: 0030312) and extracellular matrix (GO: 0031012) (Figure 2b), and significantly enriched pathways included natural killer cell-mediated cytotoxicity, protein digestion and absorption, cytokine–cytokine receptor interaction, ECM–receptor interaction, and cell adhesion molecules (Figure 2c). GSEA results showed that adhesion-related functional terms, including cell adhesion molecule binding (GO: 0050839), epithelial cell–cell adhesion (GO: 0090136), cell–cell adhesion mediated by cadherin (GO: 0044331), cell adhesion mediated by integrin (GO: 0033627), and cell–matrix adhesion (GO: 0007160), were significantly suppressed in high-altitude yak lung tissues (Figure 2d). This enrichment result suggests that the high-altitude environment may induce adaptive changes in yak lung tissues to hypoxia by affecting the activity of these biological processes and pathways, thereby enhancing their adaptation to high-altitude conditions.

### 2.3. Determination of Ca^2+^ and Mg^2+^ Concentration

**Figure 3:**
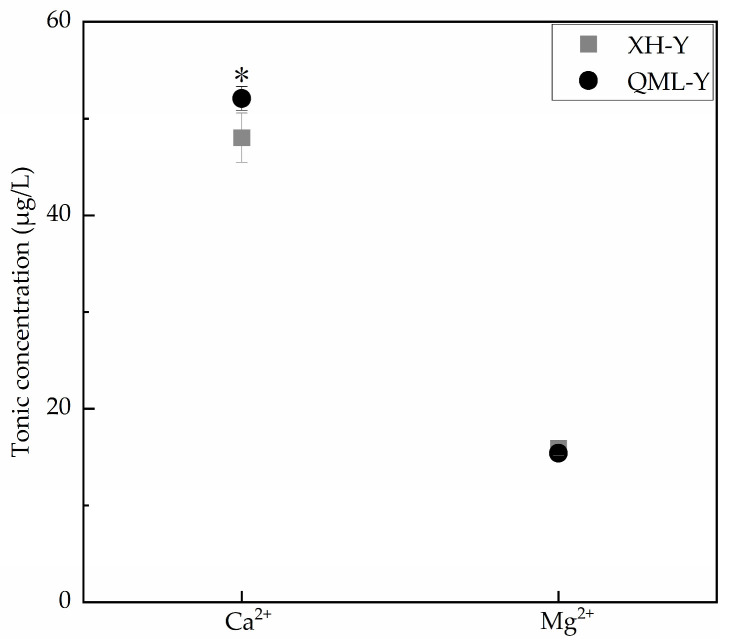
Measurement results of Ca^2+^ and Mg^2+^ concentrations in high- and low-altitude yak lung tissues. Asterisks (*) indicate statistically significant differences between groups (*p* < 0.05).

Ca^2+^ and Mg^2+^ are both critical substances for maintaining cellular functions, and their concentration variations within reasonable ranges can positively regulate cell adhesion capacity [24,25]. Inductively coupled plasma mass spectrometry was used to determine the concentrations of Ca^2+^ and Mg^2+^ in diluted samples under different altitude conditions. Results showed that compared with low-altitude yaks, Ca^2+^ concentration was significantly increased in high-altitude yak lung tissues (*p* < 0.05), while Mg^2+^ concentration showed no significant change (Figure 3).

### 2.4. Relative Expression Levels of Cell Adhesion-Related Genes

**Figure 4:**
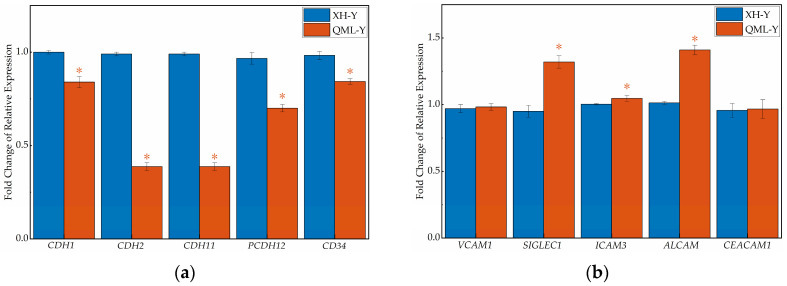

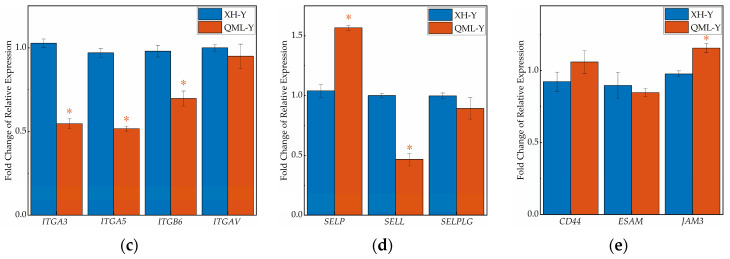
RT-qPCR results of cell adhesion-related genes in high- and low-altitude yak lung tissues. (**a**) cadherin family genes; (**b**) immunoglobulin superfamily genes; (**c**) integrin family genes; (**d**) selectin family genes; (**e**) other cell adhesion-related genes. Asterisks (*) indicate statistically significant differences between groups (*p* < 0.05).

qRT-PCR revealed that the gene expression levels of five major CAMs categories differed (Figure 4). In the lung tissues of high-altitude yaks, the relative expression levels of genes associated with the cadherin family (*CDH1*, *CDH2*, *CDH11*, *PCDH12*, *CD34*), integrin family (*ITGA3*, *ITGA5*, *ITGB6*), selectin family (*SELL*), and other adhesion molecules (*JAM3*) were significantly lower than those in low-altitude yaks (*p* < 0.05), while genes of the immunoglobulin superfamily (*SIGLEC1*, *ICAM3*, *ALCAM*) showed adaptively upregulated expression (*p* < 0.05). Additionally, no significant differences were observed in the relative expression of some CAM-related genes (*VCAM1*, *CEACAM1*, *ITGAV*, *SELPLG*, *CD44*, *ESAM*) between altitude groups.

### 2.5. Relative Expression Levels of Cell Adhesion-Related Proteins

**Figure 5:**
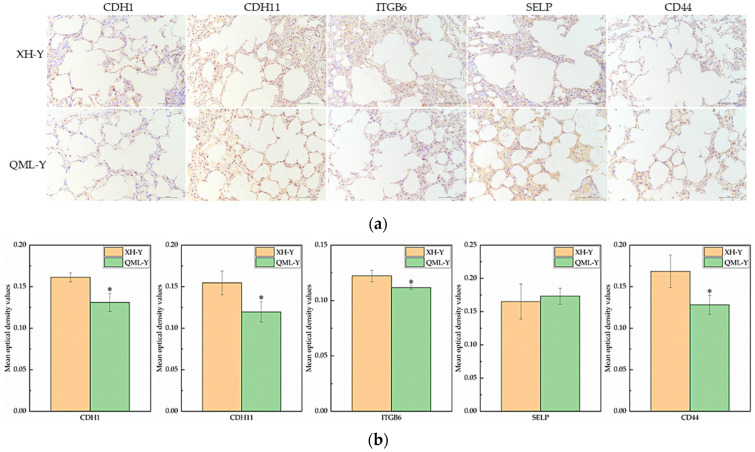
IHC staining and quantitative analysis of cell adhesion-related proteins in high- and low-altitude yak lung tissues. (**a**) IHC staining results of yak lung tissues from different altitudes, (scale bar = 100 μm); (**b**) difference analysis of mean optical density values in positively stained regions of target proteins in yak lung tissues at different altitudes, where asterisks (*) indicate statistically significant differences between groups (*p* < 0.05).

IHC staining of major cell adhesion-related molecules in yak lung tissues showed that positive signals were primarily localized on the cell membrane surface (Figure 5a). Quantitative analysis of the mean optical density in positive regions revealed that among five key adhesion proteins, CDH1, CDH11, ITGB6, and CD44 were significantly lower in high-altitude yak lung tissues compared to low-altitude yaks (*p* < 0.05), while SELP was higher in high-altitude yaks (Figure 5b).

### 2.6. Analysis of Cell Adhesion-Related Subpopulations

**Figure 6:**
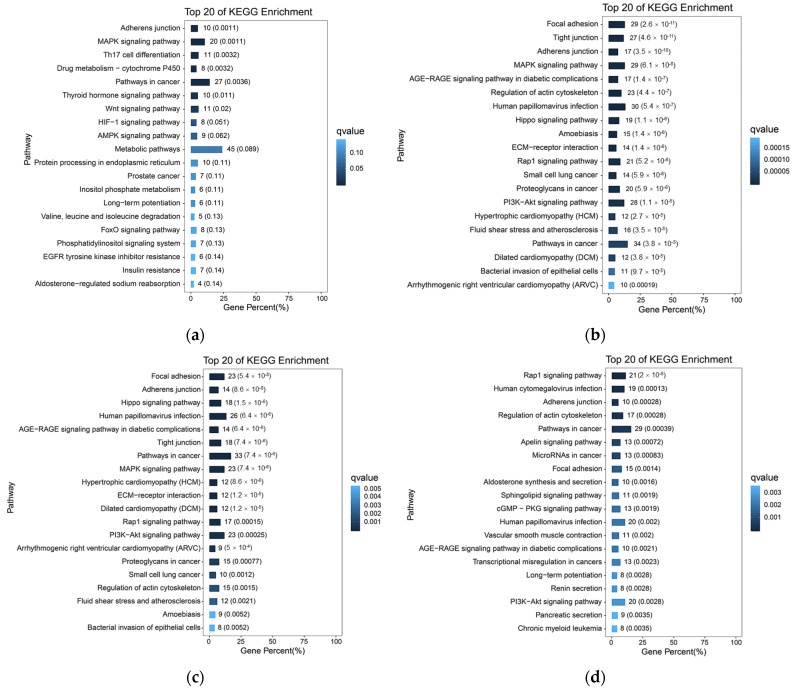

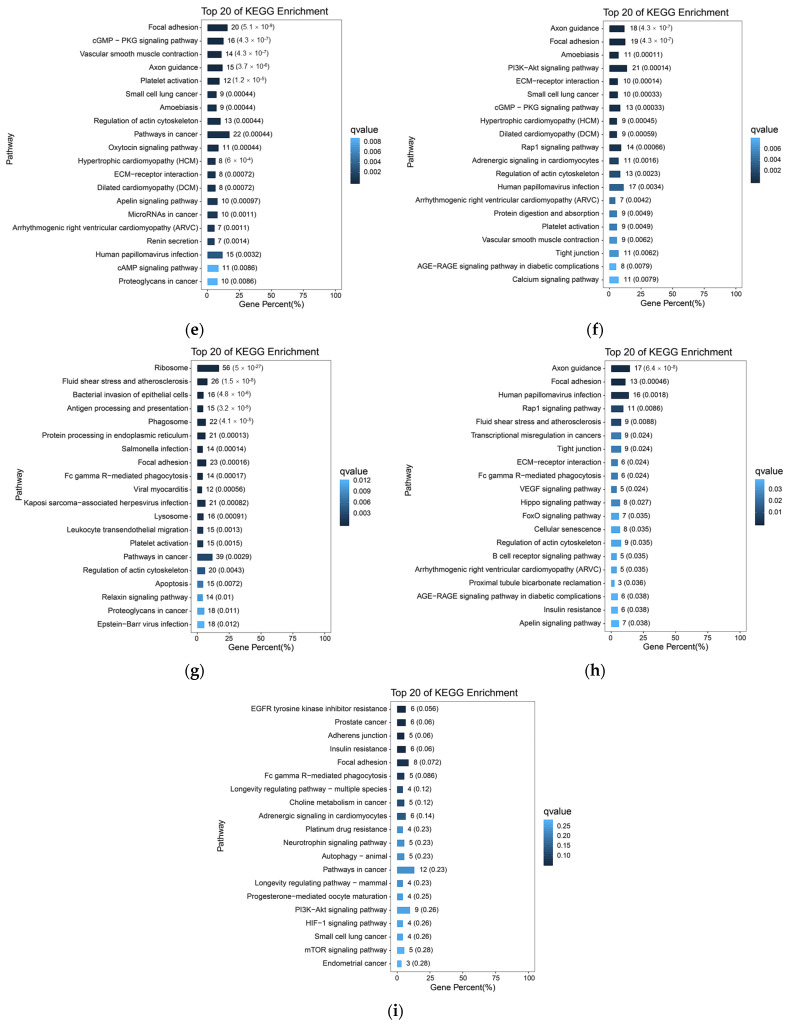
KEGG enrichment analysis results of cell adhesion-related subpopulations. (**a**) AEC II; (**b**) AEC I; (**c**) ciliated epithelial cells; (**d**) endothelial cells; (**e**) smooth muscle cells; (**f**) fibroblasts; (**g**) dendritic cells; (**h**) lymphatic endothelial cells; (**i**) basal cells.

**Figure 7:**
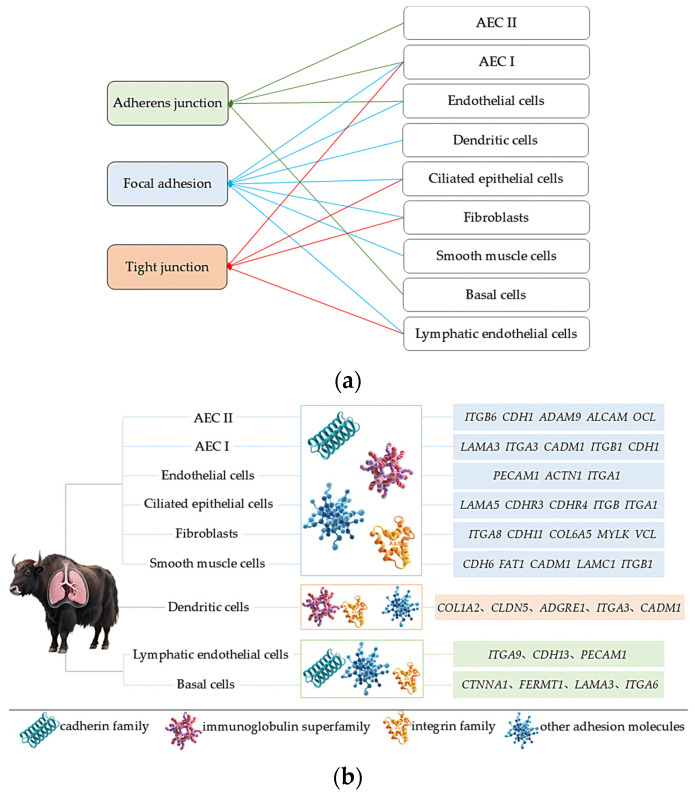
Enriched pathways, CAM types, and the identification of highly expressed genes in adhesion-related cell subpopulations. (**a**) Enrichment of DEGs from nine adhesion-related cell subpopulations in three major adhesion pathways; (**b**) identification of enriched CAM types and their highly expressed genes in nine adhesion-related cell subpopulations.

Based on the previous analysis of 10× scRNA-seq results of yak lung tissues, which identified 15 cell subpopulations [26], we further conducted GO and KEGG enrichment analyses of DEGs in all cell subpopulations from high- and low-altitude yaks. The results showed that nine cell subpopulations exhibited enrichment in cell adhesion, specifically Alveolar epithelial cells type II (AEC II), Alveolar epithelial cells type I (AEC I), endothelial cells, dendritic cells, ciliated epithelial cells, fibroblasts, smooth muscle cells, lymphatic endothelial cells, and basal cells (Figure 6). Moreover, in these nine cell subpopulations, the DEGs were primarily enriched in the adherens junction, focal adhesion, and tight junction pathways. These three pathways work cooperatively to jointly regulate cell adhesion capacity (Figure 7a). Further screened cell adhesion-related genes in these nine cell subpopulations and investigated their functions, identifying the types of CAMs that were involved. Based on the expression patterns of related genes across cell subpopulations, we selected those showing high expression levels. The results showed that all cell subpopulations in yak lung tissues were associated with multiple types of CAMs, all of which involved the integrin family (Figure 7b).

## 3. Discussion

The cell adhesion system can resist external forces that promote cell separation by enhancing adhesion capacity [27]. Our previous study on yak and cattle lung tissues from the same altitude also found that the number of cells isolated from lung tissues was influenced by cell adhesion capacity, where lower adhesion capacity facilitated cell separation [28]. This study employed two different enzymatic digestion treatments on lung tissues from yaks from different altitudes for varying durations. The cell-counting results consistently showed higher cell counts in the high-altitude group compared to the low-altitude group, with both enzymes demonstrating significant differences in cell numbers at 2 h of digestion. These findings indicate that yak lung tissues from different altitude environments exhibit different cell adhesion capacities, suggesting that high-altitude yak lung tissue cells possess weaker intercellular adhesion capacity than their low-altitude yaks. Cell adhesion capacity serves as a critical factor in maintaining tissue structure and homeostasis. It regulates cell morphology, migration, and growth, while participating in various essential physiological processes, including tissue development, barrier function establishment, intercellular communication, and immune-inflammatory responses [29,30]. Therefore, high-altitude yak lung tissue may influence immune cell activity and proper localization by regulating the strength of intercellular adhesion capacity, thereby modulating the tissue’s anti-damage repair capability and adaptively responding to stronger hypoxic stimulation. After further performing GO and KEGG enrichment analyses on the RNA-seq results of high- and low-altitude yak lung tissues, we found that DEGs were significantly enriched in functional items and signaling pathways such as immune response, ECM, and CAMs. ECM, as a dynamic three-dimensional network composed of macromolecules, provides structural support for tissues and participates in the regulation of cellular behavior [31]. CAMs mediate interactions both between cells and the ECM, playing crucial roles in tissue structure construction and maintenance, immune cell migration, and the regulation of inflammatory responses [23,32]. In high-altitude environments, yak lung tissue may enhance its structural stability and immune defense capabilities by influencing cell–ECM adhesion, thereby facilitating the yak’s adaptive survival under extreme conditions. GSEA analysis revealed that the cell adhesion function in yak lung tissues was significantly suppressed under high-altitude conditions. Any factors affecting the organism’s physiological functions must maintain reasonable expression levels, which is crucial for ensuring organismal homeostasis and particularly vital during high-altitude adaptation. CAMs, as key regulators of physiological functions in animal tissues, mediate an appropriate adhesion capacity that is crucial for maintaining organismal health [33,34]. The suppressed state of cell adhesion capacity under high-altitude conditions may represent an adaptive balancing strategy developed by yak lung tissue to cope with extreme environments. We hypothesize that moderate suppression helps maintain both tissue structural stability and the dynamic equilibrium of the immune microenvironment.

CAMs comprise five main types, each with structural and functional specificity [17]. qRT-PCR and IHC staining results showed that the expression of both cadherin family genes and integrin family genes was significantly downregulated in high-altitude yak lung tissues. Within the cadherin family, *CDH1*, *CDH2*, and *CDH11* play crucial roles in cell recognition, tissue barrier formation, and organ homeostasis maintenance [35,36]; *PCDH12* participates in key neurodevelopmental processes including neuronal maturation, dendrite formation, and axon guidance [37]; *CD34* serves as a marker for hematopoietic progenitor cells and regulates the differentiation, proliferation, and migration of hematopoietic cells [38]. Within the integrin family, *ITGA3* mediates cell–ECM adhesion [39], while *ITGA5* and *ITGB6* participate in immune cell aggregation and migration, as well as the regulation of inflammatory responses [40,41]. The moderate upregulation of CAMs’ expression facilitates tissue homeostasis maintenance and functional execution, whereas excessive expression may induce pathological alterations through abnormal intercellular interactions. Studies have shown that the overexpression of endothelial CAMs (e.g., E-selectin) can exacerbate inflammatory responses by promoting inflammatory cell infiltration [42]. Under high-altitude environments, yak lung tissue may downregulate the expression of certain adhesion genes to reduce adhesion strength, thereby maintaining normal tissue function and ensuring organismal health. Furthermore, CAMs-mediated adhesion processes are energy-dependent. The expression of certain cell adhesion-related genes is driven by acetyl-CoA, and the ACLY (ATP–citrate lyase)-mediated synthesis of acetyl-CoA can enhance cell–ECM adhesion [43,44]. Under high-altitude environments, yak lung tissue may downregulate the expression of specific CAMs to reduce the energy consumption required for maintaining cellular adhesion, thereby alleviating hypoxia-induced metabolic stress and ensuring adequate energy supply for fundamental physiological functions. Notably, immunoglobulin superfamily-related genes exhibit adaptive upregulation with increasing altitude. In addition to their roles in cell adhesion, members of the immunoglobulin superfamily primarily perform diverse regulatory functions within the immune system [45]. *SIGLEC1* facilitates pathogen clearance through the recognition of sialic acid-containing glycostructures [46], *ICAM3* mediates the identification and clearance of apoptotic cells by phagocytes [47], and *ALCAM* participates in T cell activation and migration processes [48]. The adaptive upregulation of these genes may enhance pathogen clearance efficiency and optimize intercellular immune communication, thereby ensuring the lung tissue maintain essential immune defense capabilities. This plays a vital protective role in yaks’ adaptation to high-altitude environments. In summary, the differentiated regulatory strategy of CAMs’ expression in yak lung tissue likely represents an adaptive equilibrium state to high-altitude environments. By moderately suppressing inflammatory responses and optimizing energy allocation, pulmonary tissue achieve effective adaptation to long-term hypoxic environments. This regulatory strategy not only maintains the dynamic balance between immune homeostasis and energy metabolism, but also ensures survival advantages under extreme high-altitude conditions.

As divalent cations that play crucial roles in organisms, Ca^2+^ and Mg^2+^ can enhance CAMs-mediated adhesion [49]. The binding of Ca^2+^ to specific sites in the extracellular domain strengthens cadherin-mediated intercellular adhesion [50], and its charge properties enhance cell adhesion activity by disrupting the electrostatic interactions between lysine residues of integrin αLβ2 and acidic phospholipids [51]. Mg^2+^ can promote osteoblast adhesion by optimizing the physicochemical properties of hydrogels [52], and can also provide a pro-adhesive microenvironment for macrophages through the formation of adhesive nanoscale assemblies [53]. Previously, this study speculated that yak lung tissue under high-altitude conditions would exhibit an overall relatively suppressed state of cell adhesion capacity. However, ion concentration measurements revealed significantly higher Ca^2+^ levels in high-altitude yak lung tissues compared to those in low-altitude yaks, with no significant difference observed in Mg^2+^ content. Insufficient adhesion strength may lead to tissue dysfunction, while elevated Ca^2+^ concentrations might partially alleviate the inhibitory effects of high-altitude environments on adhesion through enhancing cellular adhesive capacity. This exquisitely regulated pattern in yak lung tissue maintains stable adhesive capacity, preventing both tissue damage from excessive adhesion and functional impairment caused by over-suppression. While ensuring structural stability and the coordinated operation of functional networks, it optimizes pulmonary tissue performance and sustains functional homeostasis in yak lungs. Furthermore, as a universal second messenger, Ca^2+^ extensively participates in regulating diverse biological processes including cell proliferation, apoptosis, migration, immune responses, substance transport, muscle contraction, neurotransmission, and gene expression, playing crucial roles in maintaining organismal viability [54,55]. The endoplasmic reticulum, as a multifunctional organelle, participates in a series of interconnected physiological processes, and the maintenance of its functional integrity requires that Ca^2+^ levels are not too low [56]. When Ca^2+^ enters the interior of mitochondria, it activates key enzymes in the tricarboxylic acid cycle; elevated Ca^2+^ concentrations promote coordinated upregulation of the entire oxidative phosphorylation mechanism, resulting in accelerated respiratory chain activity and increased ATP production [57,58]. In summary, beyond enhancing cellular adhesive capacity, Ca^2+^ performs diverse functions. The measured elevation in overall Ca^2+^ concentration in high-altitude yak lung tissue is likely the integrated outcome of the participation of Ca^2+^ in regulating multiple biological processes.

Among the 15 cell subpopulations analyzed, we identified adhesion-related alterations in 9 subsets, primarily involving three pathways: adherens junctions, focal adhesions, and tight junctions. Adherens junctions are the most prevalent form of intercellular adhesion, participating in maintaining tissue architecture, regulating cellular polarity, and effectively constraining cell migration and proliferation [45]. Focal adhesions serve as bridges connecting cells to the ECM, playing pivotal roles in cell migration, proliferation, differentiation, survival, and gene expression regulation [46,59]. Tight junctions occur at epithelial connections, not only regulating the formation of epithelial and endothelial barriers along with the paracellular diffusion of ions and solutes while restricting lipid diffusion, but also transmitting signals among the cytoskeleton, nucleus, and various cell adhesion complexes [60,61]. These three pathways act synergistically to modulate cell adhesion capacity in yak lung tissue, thereby helping maintain tissue structural stability and normal physiological functions. Further analysis revealed that all cell subpopulations in yak lung tissues are associated with the integrin family. Integrins can adhere to nearly all ECM components and serve as the critical link between the ECM and cytoskeleton, while regulating multiple essential physiological processes via bidirectional intercellular signaling [62]. Hypoxia can induce varying degrees of lung tissue damage. Integrins not only drive pulmonary vascular remodeling and maintain airway epithelial integrity, but also regulate immune and inflammatory responses, playing essential roles in lung tissue repair and homeostasis maintenance [21]. Therefore, integrins are critically important for yak lung tissue’s adaptation to hypoxic environments. scRNA-seq revealed adhesion-related changes in nine distinct cell subpopulations within yak lung tissue, indicating that adaptation to high-altitude hypoxia involves a multicellular regulatory strategy. The expression of adhesion molecules in AEC I, AEC II, and endothelial cells directly supports tissue barrier stability, whereas in immune cells such as dendritic cells, it may modulate immune responses. Regarding fibroblasts, studies highlight the crucial role of fibroblast–epithelial cell interactions in maintaining pulmonary homeostasis, injury repair, and remodeling [63]. Moreover, alveolar epithelial cells are surrounded by fibroblasts and other cell types, which influence epithelial barrier function and regeneration through paracrine signaling [64]. Therefore, we speculate that adhesion-related molecules in fibroblasts and other cells may not primarily form tight junctions themselves, but rather indirectly regulate adjacent epithelial barrier function, thereby contributing to tissue microenvironment homeostasis. The differential expression of cell adhesion-related genes across multiple cell types in yak lung tissues demonstrates that CAMs play an important role in high-altitude hypoxia adaptation, with the integrin family likely serving as key modulators in this process. The adhesion changes across different cell types further reflect a complex and precise multicellular coordinated regulatory strategy for hypoxia adaptation in yak lung tissue. This multicellular coordinated adhesion regulatory network not only maintains the structural stability of lung tissue, but also ensures the functional homeostasis of lung tissue in high-altitude environments through the precise regulation of key physiological processes, including immune responses, inflammatory reactions, and tissue repair. The adhesion changes in different cell types possess unique functional features, which warrant further investigation in subsequent studies.

## 4. Materials and Methods

### 4.1. Experimental Animals and Sample Handling

This study utilized a total of six clinically healthy adult male yaks, aged approximately 3–5 years. The yaks were divided into two groups based on altitude differences in their living environments. The high-altitude experimental group consisted of yaks from Qumalai County, Qinghai Province (altitude 4500 m), designated as QML-Y (*n* = 3). The low-altitude control group consisted of yaks from Xunhua County, Qinghai Province (altitude 2600 m), designated as XH-Y (*n* = 3). All individuals were randomly selected. After yaks were slaughtered, tissue samples from the left pulmonary diaphragmatic lobe were immediately collected, followed by sequential rinsing with 0.9% saline, 75% ethanol, and 1× PBS buffer to remove residual blood cells. After dissection into small pieces, some tissue was fixed in 4% paraformaldehyde in a 15 mL centrifuge tube, while the remainder was placed in a 2 mL sterile cryovial and snap-frozen in liquid nitrogen. This research protocol was reviewed and approved by the Animal Science and Technology Ethics Committee of Qinghai University in Xining, Qinghai Province, China (Approval No.: PJ-2023037) on 15 March 2023. All experiments strictly adhered to the relevant regulations of the ‘Guidelines for the Welfare and Ethical Review of Laboratory Animals’ (GB/T 35892-2018) of the People’s Republic of China [65].

### 4.2. Isolation of Lung Tissue Cells

Cell isolation experiments from yak lung tissue were performed under sterile conditions in a biosafety cabinet, using fresh yak lung tissues as experimental material. Lung tissue devoid of fascia was minced in a sterile Petri dish containing PBS with dual antibiotics. The minced tissue was then completely transferred to a 15 mL centrifuge tube, digested with 4–5 volumes of enzyme (relative to tissue volume) at 37 °C. This study employed 0.25% trypsin (BOSTER Biological Technology, Wuhan, China) and 2 mg/mL collagenase type I (Solarbio Science & Technology Co., Ltd., Beijing, China) to digest the lung tissues, separately. The experiment was set up with three different digestion times of 1 h, 2 h, and 3 h. Upon reaching the target digestion time, filter the mixture in the centrifuge tube and collect the filtrate into a fresh tube, then terminate digestion by adding culture medium (15% Fetal Bovine Serum (ExCell Bio, Shanghai, China), 2% double antibiotics (BOSTER Biological Technology, Wuhan, China), 1% 100× ITS liquid medium supplement (Coolaber, Beijing, China), 50 ng/mL Epidermal Growth Factor (Sigma-Aldrich, St. Louis, MO, USA), and DMEM-HG medium). Following centrifugation at 1500 rpm for 5 min, the red pellet observed at the tube bottom represented erythrocytes. After supernatant removal, 3 mL of red blood cell lysis buffer (Solarbio Science & Technology Co., Ltd., Beijing, China) was added to eliminate them. After standing, centrifuge again under identical conditions, and the white pellet observed at the tube bottom represents isolated cells from the tissue. After supernatant removal, add 3 mL of culture medium and gently pipette to disperse the cells evenly, then transfer to a culture flask. Observe under an inverted fluorescence microscope for imaging and cell-counting.

### 4.3. RNA-Seq

Total RNA was extracted from yak lung tissues and mRNA was enriched using Oligo(dT) magnetic beads. Fragmented mRNA served as the template for first-strand cDNA synthesis in an M-MuLV reverse transcriptase system, followed by synthesis of the second-strand cDNA using dNTPs as substrates in the DNA polymerase I system. After the purified double-stranded cDNA underwent end repair, A-tailing, and adapter ligation, cDNA fragments of approximately 200 bp were selected for PCR amplification and product purification, finally completing the library construction. After the library quality was verified, paired-end sequencing was performed on the Illumina HiSeq 2500/4000 platform to obtain raw reads. Quality control of raw reads was performed using fastp [66] to filter out low-quality data and obtain clean reads, which were then aligned to the reference genome (BosGru_v2.0) to ensure accurate sequence alignment. Transcript assembly was performed using StringTie [67], followed by gene expression levels with RSEM, using normalized FPKM values for quantitative analysis. DEGs were defined by |fold change| ≥ 2 with FDR < 0.05. Functional enrichment analysis was performed using the GO annotation system (http://www.geneontology.org (accessed on 18 January 2024)) and KEGG pathway database (https://www.genome.jp/kegg/ (accessed on 18 January 2024)), and GSEA [68] was further used to validate activation states of relevant terms or pathways. A normalized enrichment score (NES) >1 indicates activation, while NES < −1 suggests suppression. In this study, the RNA-seq experiments for yak lung tissues from high- and low-altitude regions were conducted by Guangzhou Gidio Biotechnology Co., Ltd. (Guangzhou, China).

### 4.4. Determination of Ion Concentrations

After freeze-drying, the target ion concentration of the yak lung tissue samples was measured. The weighing dishes were dried in a 60 °C oven and numbered after cooling. Approximately 10 g of lung tissue per experimental animal was weighed, minced, placed in a weighing dish and sealed with perforated plastic wrap. After pre-cooling at −80 °C for 20 min, samples were transferred to a pre-chilled freeze-dryer for ~36 h until complete lyophilization. The freeze-dried samples were equilibrated to room temperature in a desiccator, then ground into powder using a grinder. About 0.1 g of powdered sample was weighed into a dedicated microwave digestion vessel, followed by the sequential addition of 7 mL HNO_3_ and 2 mL H_2_O_2_. After overnight standing, the microwave digestion program was executed. Post-digestion, the cooled digestate was transferred to an acid evaporation system for acid removal treatment at 170 °C for ∼130 min. After acid removal, the vessel and lid were rinsed with ultrapure water, and the solution was diluted to 50 mL in a volumetric flask. Finally, after 1000-fold dilution, Ca^2+^ and Mg^2+^ concentrations were determined by inductively coupled plasma mass spectrometry.

### 4.5. qRT-PCR

Cell adhesion-related genes in yak lung tissues were screened, and their CDS sequences were retrieved from the NCBI database (https://www.ncbi.nlm.nih.gov/ (accessed on 15 February 2024)). Gene-specific primers were designed using Oligo 7.0 software, and their specificity was validated by qRT-PCR amplification curves and agarose gel electrophoresis to ensure experimental reliability. For qRT-PCR analysis of these genes, *GAPDH* served as the internal reference. The experimental process primarily included total RNA extraction, RNA reverse transcription to cDNA, qRT-PCR reaction mixture preparation, and qRT-PCR amplification. Relative gene expression was calculated using the 2^−ΔΔCt^ method. Specific primer sequences and detailed protocols were referenced from published studies [28].

### 4.6. IHC Staining

IHC staining detects target protein expression in tissue sections through antigen–antibody-specific binding [69]. Yak lung tissue samples were processed using the formalin-fixed paraffin-embedding method, sectioned with a microtome (Shanghai Leica Instrument Co., Ltd., Shanghai, China), and mounted on glass slides. Following the dewaxing and rehydration of paraffin sections, antigen retrieval (citrate buffer, pH 6.0), endogenous peroxidase blocking (3% hydrogen peroxide solution), and serum blocking (3% BSA) were performed sequentially. In this study, the secondary antibodies and primary antibodies against CDH1 and CD44 were sourced from Wuhan Servicebio Technology Co., Ltd. (Wuhan, China), while the primary antibodies targeting CDH11, ITGB6, and SELP were obtained from Thermo Fisher Scientific (Waltham, MA, USA). On different sections, the following antibodies were added at a dilution ratio of 1:500: anti-E-cadherin (CDH1) mouse monoclonal antibody (Cat# GB12083-100), anti-cadherin-11 (CDH11) mouse monoclonal antibody (Cat# 32-1700), anti-integrin β6 (ITGB6) mouse monoclonal antibody (Cat# MA5-23968), anti-P-selectin (SELP) rabbit polyclonal antibody (Cat# PA5-79973), and recombinant anti-CD44 mouse monoclonal antibody (Cat# GB152054-100), followed by overnight incubation at 4 °C. After washing with PBS, the primary antibody-treated sections were added with goat anti-mouse IgG labeled by HRP (Cat# GB23301) or HRP-labeled goat anti-rabbit secondary antibody (Cat# GB23303) at a 1:200 dilution ratio, followed by incubation at room temperature for 50 min. Diaminobenzidine staining visualized brown-yellow positive signals, while cell nuclei stained with hematoxylin appeared blue. The chromogenic reaction was terminated by rinsing with tap water. Subsequently, standard dehydration and mounting procedures were performed. The stained tissue sections were observed under a Nikon microscope. Within the alveolar regions, six non-overlapping fields of view (200× final magnification) were systematically and randomly selected while avoiding blood vessels, and images were captured using a DP70 microscopic image acquisition system (Olympus, Tokyo, Japan).

### 4.7. scRNA-Seq

scRNA-seq can precisely resolve the transcriptomic characteristics of individual cells, revealing their functional states, cellular heterogeneity, and gene regulatory networks within their microenvironment [70,71]. The experimental procedures, raw data processing, and classification/identification criteria for cell subpopulations are described in published studies [72]. DEGs between different cell subpopulations in yak lung tissue were identified using the conditions of *p* ≤ 0.01 and |log2(Fold Change)| > 0.585. The expression levels of these genes must exceed 25% in all relevant cell subpopulations and control subpopulations [73]. GO and KEGG enrichment analyses were performed on DEGs to screen significantly enriched cell adhesion-related terms and pathways. Subsequently, adhesion-related genes were further selected and combined with functional annotation results for cell subpopulation localization, identifying key cell types involved in adhesion functions in yak lung tissue. Within adhesion-functional cells, genes associated with adhesion were screened, and their functions were queried based on the literature, the NCBI database, and the GeneCards database (https://www.genecards.org/ (accessed on 23 March 2024)) to determine the CAM types in each subpopulation.

### 4.8. Data Analysis

Cell-counting and a quantitative analysis of IHC staining were performed using Image-Pro Plus 6.0 software. For IHC quantification, a uniform color threshold was first applied to accurately identify DAB-positive (brown-yellow) signals, after which the positive staining area (Area) and integrated optical density (IOD) in each field of view were measured, and the optical density (IOD/Area) was finally calculated. A statistical analysis of experimental data was conducted with SPSS 25.0, employing independent samples *t*-tests to evaluate intergroup differences. Results were expressed as mean ± standard deviation (x^-^ ± SD), with statistical significance set at *p* < 0.05. Data visualization was accomplished using Origin 2024 software. The visualization of DEGs enrichment results from scRNA-seq data was generated with the ggplot2 package in R software (version 4.2.1).

## 5. Conclusions

Our research found that yak lung tissue maintains moderate cell adhesion capacity under different altitude conditions through finely regulated CAMs expression, thereby ensuring structural stability and coordinated functional network operation while achieving dynamic equilibrium between immune regulation and energy allocation. Under high-altitude conditions, yak lung tissue downregulates certain CAMs expression to prevent tissue damage from excessive adhesion, while adaptively upregulating certain CAMs to avoid functional impairment caused by insufficient adhesion. This differential regulatory strategy of CAMs reflects an adaptive equilibrium state in yak lung tissue responding to high-altitude environments, achieving a dynamic balance of adhesive capacity across different altitude conditions. Furthermore, integrin family members were identified in all nine adhesion-related cell subpopulations, suggesting their pivotal role in regulating hypoxia adaptation in yak lung tissue. In summary, the differential expression of CAMs is an important regulatory strategy for yak lung tissue to adapt to intensified hypoxic stress at higher altitudes.

## Data Availability

The sequence data for this study is available at NCBI SRA: PRJNA1127767.
